# Impact of soil contamination on the growth and shape of ant nests

**DOI:** 10.1098/rsos.180267

**Published:** 2018-07-11

**Authors:** Jean-Baptiste Leclerc, Jennifer Pinto Silva, Claire Detrain

**Affiliations:** Unit of Social Ecology, Université Libre de Bruxelles, CP 231, Boulevard du Triomphe, 1050 Brussels, Belgium

**Keywords:** social immunity, soil contamination, nest topology, *Myrmica* ants, *Metarhizium* fungus

## Abstract

As entomopathogens are detrimental to the development or even survival of insect societies, ant colonies should avoid digging into a substrate that is contaminated by fungal spores. Here, we test the hypotheses that *Myrmica rubra* ant workers (i) detect and avoid fungus-infected substrates and (ii) excavate nest patterns that minimize their exposure to entomopathogenic spores. Small groups of *M. rubra* workers were allowed to dig their nest in a two-dimensional sand plate of which one half of the substrate contained fungal spores of *Metarhizium brunneum*, while the other half was spore-free. We found that the overall digging dynamics of *M. rubra* nests was not altered by the presence of fungus spores. By contrast, the shape of the excavated areas markedly differed: control nests showed rather isotropic patterns, whereas nests that were partially dug into a fungus-contaminated substrate markedly deviated from a circular shape. This demonstrates that the sanitary risks associated with a digging substrate are key factors in nest morphogenesis. We also found that *M. rubra* colonies were able to discriminate between the two substrates (fungus-infected or not). Furthermore, some colonies unexpectedly showed a high consistency in excavating mainly the infected substrate. This seemingly suboptimal preference for a contaminated soil suggests that non-lethal doses of fungal spores could help ant colonies to trigger ‘immune priming’. The presence of fungi may also indicate favourable ecological conditions, such as humid and humus-rich soil, that ants use as a cue for selecting suitable nesting sites.

## 1. Introduction

Insect societies have developed a complex network of social interactions that allow them to achieve efficient cooperation for the benefit of the whole colony. These patterns of interactions are shaped, at least partially, by the spatial structure of the nest. Nest patterns result from stigmergic processes in which the built structure acts as a feedback on the digging behaviour of individuals, leading to adaptive, self-organized patterns, without the need for any template, centralized control or even direct communication between nest-mates [1–3]. Although composed of basic building modules such as chambers interconnected by galleries, the nest patterns of insect societies can be highly diverse, from non-ramified to tree-like structures [4]. These structures can be modified in order to meet specific functional values such as thermoregulation [5], food storage [6], nest ventilation [7] or protection against intruders and predators [8]. Furthermore, by spatially organizing interactions between workers, the nest acts as an effective barrier against disease transmission [9,10]. In particular, the nest structure can limit contact between pathogen-exposed foragers located in the peripheral nest chambers and inner-nest individuals, such as larvae or queens [9]. Seen in this way, the nest pattern can be considered as part of ants' social immunity, a term describing the colony-level disease protection resulting from cooperation between nest-mate individuals [11]. As the location and the structure of collectively built nests determine the level of ants’ exposure to soil pathogens, the digging patterns should be considered as organizational components of social immunity that, together with behavioural and physiological adaptations, prevent pathogen uptake and transmission in the ant colony.

The topological features of a nest reflect both the intrinsic features of the colony and the characteristics of its environment. In the case of subterranean nests built by social insects, colony size [2,4,12,13] and environmental factors, such as humidity [14], temperature [15] or granulometry of the digging substrate [16], regulate the digging behaviours of individuals and thus the final shape of their nest. The contamination of the soil by pathogens can also influence the digging activity of termites and ants. For instance, *Coptotermes lacteus* termites display an avoidance response or dig out shorter tunnels into substrates infected by *Metarhizium brunneum* fungus [17]. Likewise, *Solenopsis invicta* ant workers selectively avoid building their nest in nematode-infected soils [18]. However, some counterexamples exist. For example, *Formica selysi* queens [19] and *Monomorium pharaonis* ant workers [20] show a strong preference for fungus-contaminated nests as opposed to spore-free nests.

In the present study, we challenged *Myrmica rubra* ant colonies with soil patches infected by *Metarhizium brunneum* spores. We investigated whether the choice of the substrate, the digging dynamics, as well as the size and topology of the nest were influenced by the presence of potentially harmful pathogens. For this, we tested small groups of 50 *M. rubra* workers in a two-dimensional digging set-up [13,16] in which one half of the substrate contained fungal spores of *Metarhizium brunneum*, while the other was spore-free. This allowed us to assess whether a contaminated substrate leads to a decrease of excavated soil as well as to a nest topology that minimizes the level of ants' exposure to fungus spores.

## 2. Material and methods

### 2.1. Maintenance of ant colonies

Eleven colonies of *M. rubra* ants containing one queen, 200–300 workers and brood were used for the experiments. In the laboratory, each colony was reared in a plastic tray (Janet type: 47 × 29 cm) in which the floor was covered with plaster and the borders were coated with polytetrafluoroethylene (Fluon, Whitford, UK) to prevent ants from escaping. A square 10 cm wide glass plate, placed 3 mm above the ground and covered with a red filter, was used as a nest ceiling. Each colony was fed with one mealworm (*Tenebrio molitor*) three times per week, while water and sucrose solution (0.3 M) were provided ad libitum. Laboratory conditions were kept at a 21 ± 1°C and 50 ± 5% humidity rate, with a constant photoperiod of 12 h per day.

### 2.2. Preparation of spore suspensions

We used a commercial strain of *Metarhizium brunneum* fungus (Strain F52 from Novozymes) that is produced in the form of barley grains coated with fungal spores. This generalist entomopathogen fungus is known to kill more than 200 insect species [21] and to prevail in the soil fungi communities of many different biotopes, including those inhabited by *M. rubra* ants [22,23]. Four barley grains were first put in a Petri dish (55 mm diameter) lined with a thin layer of potato dextrose agar (Sigma-Aldrich). The dish was then placed in an incubator for 14 days at a temperature of 25°C to provide optimal conditions for sporulation. Fresh conidia were then collected in 5 ml of 0.05% Triton-X solution (Sigma-Aldrich) [24]. We estimated spore concentration by counting spores on a haemocytometer (1 µl) placed under a microscope (400× magnification). Finally, dilutions were made using 0.05% Tween 20 (Sigma-Aldrich) to reach a final concentration of around 1 × 10^6^ spores ml^−1^. In addition, the viability of conidia was determined by placing 5 ml of the final solution of spores on a thin layer of potato dextrose agar and by incubating it at 25°C for 4 days.

### 2.3. Preparation of digging substrates

We used Brusselian sand as a digging substrate. This sand has the great advantage of having a fine and homogeneous granularity that prevents the nest from collapsing during the digging process. Before being used as a nest substrate, the sand was sieved and sterilized at a temperature of 100°C for 45 min. We then prepared the fungus-contaminated substrate by mixing 25 ml of the spore solution (1 × 10^6^ spores ml^−1^) per 100 g of sand. As a control, spore-free substrate was made by adding 25 ml of solution containing 0.05% Tween 20 and 0.05% Triton-X per 100 g of sterilized sand*.* The used level of *Metarhizium* spores (25 × 10^4^ spores per g soil) was of the same order of magnitude as the natural density of *Metarhizium* detected in soils (on average 10^3^–10^4^ CFU g^−1^ soil reported by Keller *et al*. [22]). By choosing the upper value of natural spore levels, we posed ant colonies with a clear-cut sanitary challenge when they dig their nest in a fungus-contaminated substrate. Based on the protocol developed by Toffin *et al*. [13], the digging area of each nest consisted of two glass plates (20 × 20 cm), between which we spread out a thin layer of sand (2 mm high, 180 g) to allow a two-dimensional view of the digging activity through time. In the case of experimental nests, the digging area consisted of two equal halves (10 × 20 cm) of infected (90 g) and spore-free sand (90 g). The digging area of control nests was made of two halves of spore-free sand.

### 2.4. Digging activity and nest pattern

From each of the 11 colonies (hereafter called mother colonies), we randomly sampled three groups of 50 workers: two groups were assigned to the experimental condition (experimental nests, *N* = 22) and the third one was used as control (control nests, *N* = 11). The replication of the experimental condition allowed us to assess the effect of the mother colony on the digging response of ants to the contaminated substrate. Each group of 50 workers was dropped into a circular arena (55 mm diameter) to be tested 2 h later and was not fed until the end of the experiment to prevent them from being engaged in other tasks than nest-excavating ones. This starvation did not reduce the ants’ survival, because less than 2% (mean: 1.84 ± 0.26%, *N *= 33) of the ants died at the end of the experiment. The nest sand plates were randomly placed in groups of four in a closed wooden box to simulate the darkness of natural nests. We started the experiment by connecting the circular arena hosting the group of 50 tested ants to a central hole made on the upper glass plate covering the digging area. The connection was made with a vertical plastic tube (3.5 cm) that was filled with clean sand to encourage ants to start digging. The digging process was followed for 40 h once the first ant reached the central hole of the nest sand plate. Snapshots of the digging area were taken under red light every 5 h using a Logitech camera (HD Pro C920) placed 20 cm below the glass plates. Image J software was used to automatically compute both the dug area (*A*) and the perimeter (*P*) of the nest for each snapshot. This allowed us to quantify the dynamics of digging activity and to compare nest patterns between the two halves of each nest plate.

### 2.5. Statistical analyses

Statistical analyses were performed by using Statistica software v. 10 (© StatSoft, Inc.). Non-parametric tests with a significance level of *α* = 0.05 were used because all data did not meet the normality assumption. With regard to the digging activity, a generalized linear mixed-effects model (GLMM) was used to investigate the effect of treatment (control versus experimental nests), colony and time on the area excavated by colonies. Colony and treatment were treated as categorical variables, whereas time was considered as a continuous variable. Moreover, time and treatment were specified as fixed effects, colony as a random effect and replicates as a nested random factor within the colony to account for the repeated measurements performed on mother colonies [25]. Full models included treatment (control or experimental nest), colony and time as explanatory variables, and time by treatment interaction. In addition, we used a Mann–Whitney *U* test to compare the final excavated volumes between control and experimental nests.

Within each type of nest, we also used GLMM analyses to test for the effect of the side (left versus right side for the control nests or clean versus infected side for the experimental nests), colony and time on the area excavated by ants. Wilcoxon matched-pairs tests were used to compare the final excavated areas between the two sides in the experimental or in the control nests. To provide evidence of ants' preference for a spore-free substrate, we tested whether the number of ant colonies for which the most dug part was the clean half of the set-up differed from random by using a binomial test.

We characterized nest patterns by their level of digitation as well as by the anisotropy of their shape. As regards the level of digitation in control or in experimental nests, we assessed whether the perimeters of the final excavated areas significantly deviated from those expected from a circular shape, by using the Wilcoxon matched-pair test. In the case of a perfect circle, the relationship between the area (*A*) and the perimeter (*P*) of a nest can be described by the linear equation log(*P*) = log(*μ*) + *ω* log(*A*), where the parameters’ values are *μ* = 2√*π* and *ω* = 0.5. For both the control and the experimental nests, the values of *μ* and *ω* were estimated from the intercept and the slope of regression lines that best fitted log-transformed values of final perimeters as a function of final areas. The slopes and intercepts of these linear fittings were compared between experimental and control nests by using *F*-tests.

**Figure 1.**
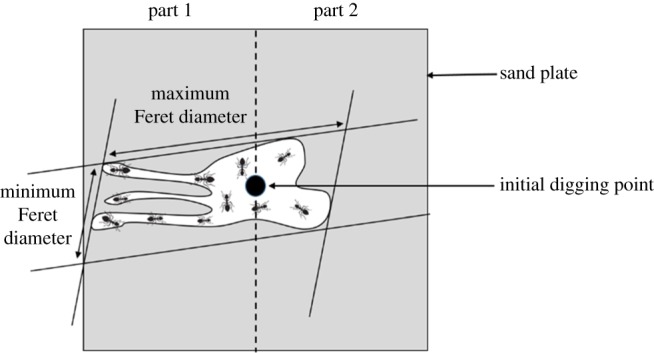
Characterization of nest anisotropy. The aspect ratio is given by the maximum over the minimum values of Feret diameters. High values of the aspect ratio characterize patterns that are strongly asymmetrical. Part 1 of the set-up was made of spore-free substrate in all the nests. Part 2 was made either of spore-free substrate in the control nests or of fungus-contaminated substrate in the experimental nests.

As regards the anisotropy of nest shapes, we first measured the maximum and the minimum Feret diameters which are, respectively, the maximal and the minimal distances between two parallel tangents of the nest shape (figure 1). These two values were calculated after considering all possible orientations of tangents (0°–180°). The aspect ratio, i.e. the maximum over the minimum Feret values, indicates the anisotropy of the pattern [26], with high aspect ratios characterizing patterns that are strongly asymmetrical. The aspect ratios were compared between control and experimental nests by using Mann–Whitney *U* tests.

To test for a colonial effect on the digging activity, we used Kendall's coefficient of concordance to assess whether experimental groups coming from the same mother colony had excavated similar nest areas at the end of the experiment. In addition, a colonial preference for one type of substrate was assessed by using the McNemar test.

### 2.6. Ethical note

No licences or permits were required for this research. Ant colonies were collected with care in the field and maintained in nearly natural conditions in the laboratory. Ants were provided with suitable nesting sites, food and water, thus minimizing any adverse impact on their welfare. After the experiments, fungal-infected ants were removed from their foraging area to protect colonies from disease spread and were killed by freezing. The rest of the colony was kept in the laboratory and reared until their natural death.

## 3. Results

### 3.1. Digging activity

**Figure 2.**
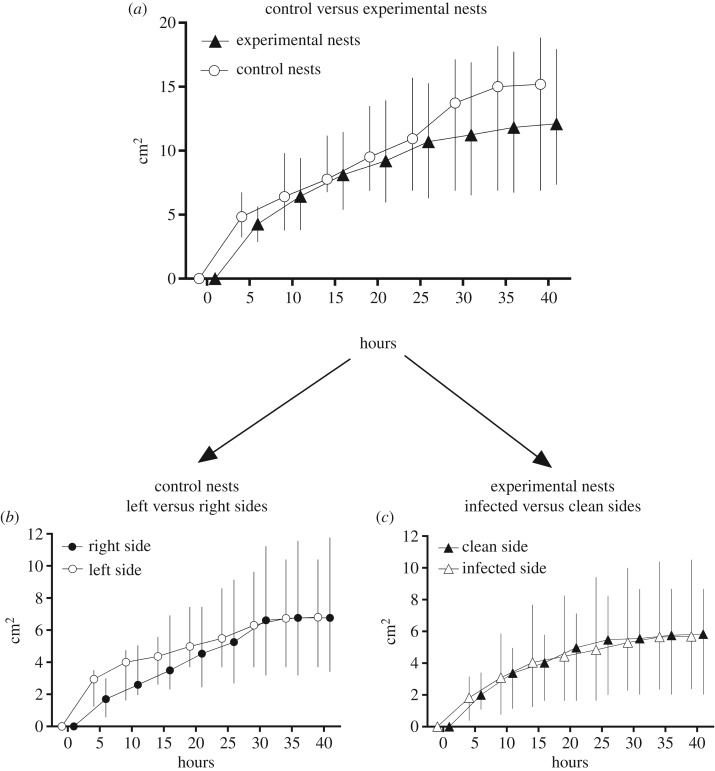
Dynamics of excavated areas (median ± interquartile range) (*a*) in control (*N* = 11) and experimental nests (*N* = 22), (*b*) in the left versus the right side of the control nests and (*c*) in the fungus-contaminated versus the spore-free side of the experimental nests.

We found no significant effect of treatment or time by treatment interaction on nest growth dynamics (GLMM: treatment effect: *F*_1.250_ = 0.02; *p* = 0.88 and time by treatment interaction: *F*_1.250_ = 0.51; *p* = 0.48; figure 2*a*). However, for both control and experimental nests, there was a highly significant effect of time (GLMM: time effect: *F*_1.250_ = 119.02; *p *< 0.001) on the excavated area. Ants were the most active in digging during the 10 first hours with around half of the final total area being excavated (mean of 42.2% and 53.1% for the control and the experimental nests, respectively; figure 2*a*). From 30 h onwards, the digging activity nearly ceased, and the excavated areas increased by only 9.9% for the control nests and by 7.5% for experimental ones during the last 10 h. Ultimately, a similar excavated area was reached in the control and experimental nests (median values of 15.2 cm² and 12.1 cm², respectively; Mann–Whitney *U* test: *U*= 90; *p* = 0.55; figure 2*a*).

In control nests, the left and the right side of the sand plates were dug with similar growth dynamics (GLMM: time by side interaction: *F*_1.162_ = 1.68; *p* = 0.20) and led to the same total area excavated (median of 6.8 cm² for both the left and the right sides; Wilcoxon matched-pair test: *T* = 28; *p* = 0.66; figure 2*b*).

Similarly, the growths of dug areas over time in the infected and the clean sides of the experimental nests were not different (GLMM: time by side interaction: *F*_1.327_ = 3.67; *p* = 0.06. figure 2*c*), and reached similar final excavated areas (median of 5.8 cm² and 5.7 cm² for the clean and the infected side, respectively. Wilcoxon matched-pair test: *T* = 95; *p* = 0.31; figure 2*c*).

**Figure 3.**
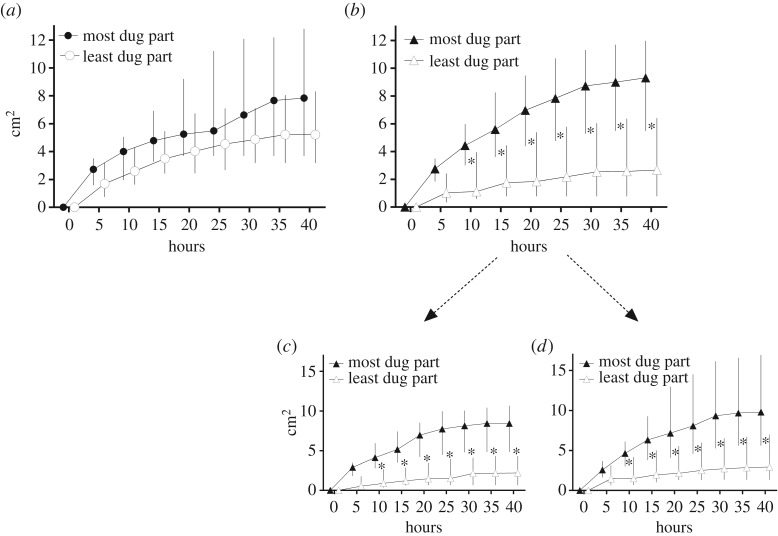
Dynamics of excavated areas (median ± interquartile range) in the most dug part (filled symbols) and in the least dug part (empty symbols) in the case of (*a*) control nests or of (*b*) experimental nests. Change in the excavated area (median ± s.d.) over time when (*c*) the clean side (*N* = 10) or (*d*) the infected side (*N* = 12) was the most dug part of the experimental nests.

With regard to the ants' preference for digging into one side of the set-up, the percentage of colonies that mostly dug in the left side (54%, *N* = 11) of control nests did not differ from random (binomial test: *p* = 0.50). This confirmed that there was no bias due to external stimuli or substrate heterogeneities in our sand plates. The most dug part of control nests grew at a rate that was only slightly faster and not significantly different from the least dug one (GLMM: time by side interaction: *F*_1.162_ = 2.42; *p* = 0.12; figure 3*a*). The findings were quite different for the experimental nests. When considering the growth dynamics in the most dug part, the excavation increased at a significantly higher rate than the least dug part of the set-up (GLMM: time by treatment interaction: *N* = 22; *F*_1.338_ = 30.48; *p *< 0.001; figure 3*b*). As a result, from 10 h onwards after the start of the digging activity, the excavated volume became significantly larger in the most dug part of the set-up compared to the other side (GLMM, Tukey's *post hoc* test: *p* = 0.006). Unexpectedly, not all the ant colonies preferred to dig into the spore-free side of the experimental nests. Indeed, the proportion of colonies (55%, *N* = 22) that had mostly dug the clean half of the set-up did not differ from random (binomial test: *p* = 0.74). Furthermore, differences in the growth dynamics between the most and the least dug side were of the same magnitude, regardless of whether the most dug part was the clean side (GLMM: time by treatment interaction: *N* = 10; *F*_1.147_ = 13.26; *p *< 0.001; figure 3*c*) or the infected side (GLMM: time by treatment interaction: *N* = 12; *F*_1.177_ = 35.58; *p *< 0.001; figure 3*d*).

### 3.2. Nest pattern

**Figure 4.**
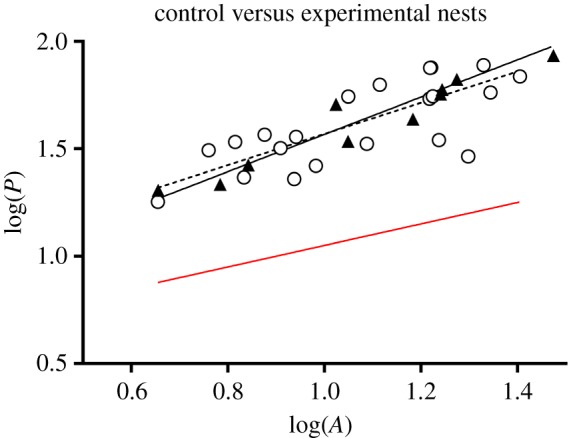
Final perimeters (*P*) as a function of final excavated areas (*A*) in control nests (*N* = 11; filled triangles) and in experimental nests (*N* = 22; empty circles). Data values were log-transformed. Then, they were best fitted by regression lines of which the equations were log(*P*) = 0.86 * log(*A*) + 0.69 (*R*^2^= 0.92, black plain line) for control nests and log(*P*) = 0.72 * log(*A*) + 0.84 (*R*^2^ = 0.59, black dashed line) for experimental nests. As a reference, the red line illustrates the expected length of perimeter as a function of the excavated area in the case of a circular shape.

Owing to their digitated contour, the perimeters of the final excavated areas were always higher than those expected from a circular shape of the same area, for both the control and the experimental nests (Wilcoxon matched-pair tests: *T* = 0; *p* = 0.003 and *T* = 0; *p *< 0.001 for the control and the experimental nests, respectively; figure 4). In addition, control and experimental nests showed the same linear relationship between the log-transformed final values of the perimeter's length and excavated area (*F*-tests: slopes: *F*_1.29_ = 0.57; *p* = 0.46; intercepts: *F*_1.30_ = 0.07; *p* = 0.79; figure 4), demonstrating that deviation from a perfect circle was of the same order of magnitude regardless of soil contamination.

**Figure 5.**
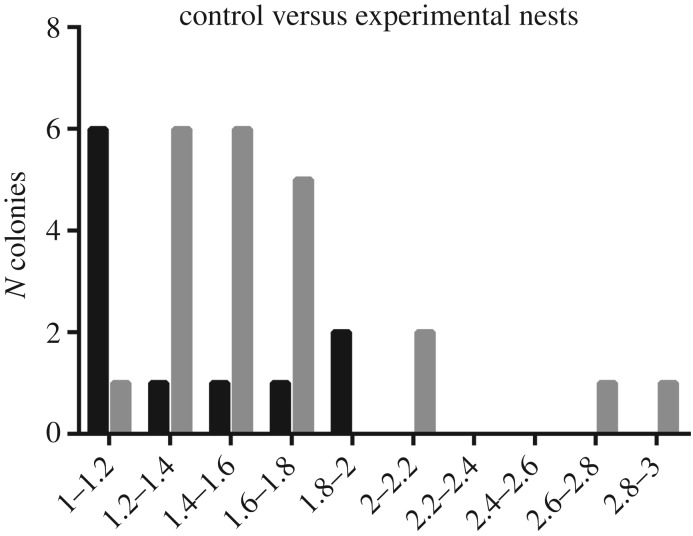
Distribution of the aspect ratios, defined as the maximum over the minimum Feret diameters, in control (*N* = 11, black bars) and experimental nests (*N* = 22, grey bars).

**Figure 6.**
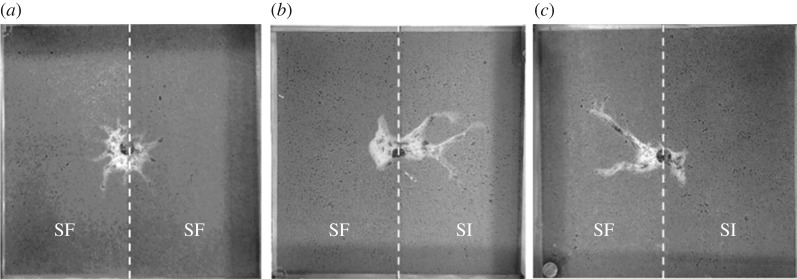
Examples of dug nests. (*a*) Control nest. The two halves of the sand plate were spore-free (SF). (*b*,*c*) Experimental nests. One half of the sand plate was spore-free (SF) and the other half was infected by *Metarhizium* fungus (SI).

The anisotropy of nest patterns was estimated by the aspect ratio, i.e. the maximum over the minimum Feret values. These ratios were significantly higher for the experimental nests than for the control ones (Mann–Whitney *U* test: *U* = 65; *p* = 0.03; figure 5). Typically, the final pattern of control nests remained rather symmetrical (figure 6*a*), while ants' digging activity seemed more directional in experimental nests, leading to the emergence of long galleries extending preferentially in one side of the set-up (figure 6*b*,*c*).

### 3.3. Colonial effect

**Figure 7.**
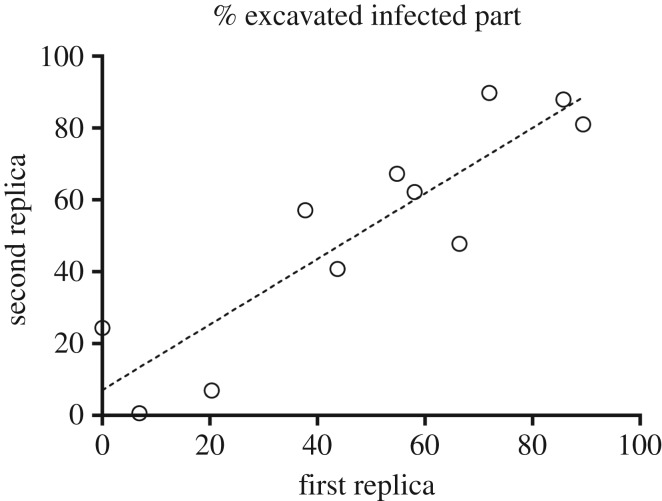
Percentage of the final nest area excavated in the infected part of the sand plate observed for the two replicates that originated from the same mother colony (*N* = 11). Data were fitted by a linear regression (*Y* = 0.91 * *X* + 7.08) (*R*² = 0.79).

We found a strong colonial effect on ants’ digging activity (GLMM: colony effects: *F*_21.327_ = 9.41; *p *< 0.001). Indeed, the final excavated areas were highly correlated between the two experimental groups that originated from the same mother colony (Kendall test: *τ* = 0.89; *p *< 0.05). Surprisingly, a colonial effect was also observed in the ants' preference for a given type of digging substrate. In most cases (nine out of 11 colonies), each pair of experimental groups that came from the same mother colony chose to focus the main part of their digging activity in the same type of substrate (McNemar test: *χ*² = 0.50; *p* = 0.48). Indeed, the percentages of the total area that were dug into the infected side of the experimental nests were highly correlated between the two replicates (figure 7).

## 4. Discussion

*Myrmica rubra* ants that were faced, at least during the first steps of digging, with a soil contaminated by fungal spores excavated nests whose patterns differed from the symmetrical shape of control nests built out of a spore-free substrate. Indeed, the presence of spores in one half of the experimental nests led to anisotropic patterns with a few galleries extending more in one side of the set-up. Surprisingly, half of the colonies preferentially dug the contaminated part of the substrate, while the other half of colonies focused excavation in the spore-free side of the set-up. In addition, this preference for one type of substrate seemed to be a colonial trait, as shown by the high consistency of choices made by groups of digging ants that were drawn from the same mother colony.

With regard to the digging activity, *M. rubra* workers were poorly efficient in nest excavation compared to other ant species such as *Lasius niger* of which the same number of workers dug out 10 times larger areas [13]. As commonly observed for several ant species [13,27,28], the digging dynamics showed a logistic shape. First, the excavation behaviour of ants was amplified by several processes such as the release of attractive digging pheromones and/or through interattraction between workers [2,13]. Then, the excavation rate progressively decreased until the nest volume was adjusted to the size of the ants’ population [4,29]. This digging dynamics is usually coupled with morphological transitions from a round to ramified shape of the excavated area [12,13,16,30]. During the first steps of nest excavation, the high density of ants along the initially short perimeter of a nest promotes a uniform digging activity and hence the round and smooth shape of the nest. Then, as the nest area increases, the average density of digging ants falls to a critical value and small buds appear on the nest perimeter at locations where the number of digging ants was still high. Finally, while the main chamber stops expanding, buds are enlarged and become lateral galleries. In other words, a high density of digging workers promotes a circular main chamber that expands in an isotropic way, whereas a low density of diggers locally promotes the formation of galleries and anisotropic nest patterns. This suggests that the higher level of anisotropy observed in our experimental nests may result from lower densities of digging ants along the nest perimeter. The lower densities of digging workers could be due to some individuals refraining from excavating and being engaged in other tasks such as increased grooming, once they were initially faced with fungal spores. Therefore, similarly to the colony size [2,12,13] or the granularity of the substrate [16], the soil contamination by a pathogen is another factor that has a considerable impact on nest morphogenesis, most probably by modifying the digging rate and/or local density of digging individuals.

Pathogen avoidance is considered a first line of disease defence in animals. In the case of social immunity, insect societies should reduce exposure to sanitary risks by avoiding digging their nest in contaminated areas. However, pathogen prevalence is quite variable inside *M. rubra* nests as specific entomopathogen groups (*Isaria fumosorosea* and nematodes) are less abundant inside nests, whereas others (*Beauveria brongniartii*) are more frequent inside than outside ant nests [31]. In sharp contrast with our expectations, around half of the colonies dug most of their nest area in the infected side of the set-up. Excavating in a contaminated substrate appears as a counterintuitive and maladaptive behaviour because *Metarhizium brunneum* fungus is known to be efficient at killing *M. rubra* workers [32–35]. These findings also contrast with previous studies reporting that insects actively avoid direct physical contact with entomopathogenic fungi [36–41], possibly by perceiving chemicals emitted by fungal spores [38,39].

Previous studies on *Atta sexdens* ants [40] and on *Macrotermes michaelseni* termites [41] showed that workers increasingly avoided fungal pathogens depending on their concentration and their virulence. In our case, the level of soil contamination could be under the threshold that enables the detection of spores by ants or that triggers ants' avoidance. However, this explanation should be discarded for the two following reasons. First, if the amount of conidia was too low to be detected by ants, the patterns of experimental nests should have been similar and as symmetrical as those of control nests in which ants were faced with a spore-free substrate. Second, because colonies were highly consistent between replicates in preferentially digging into either the infected or the spore-free sand, this strongly suggests that ant workers were able to discriminate between the two substrates. In half of the tested colonies, nest-mates even seemed to be attracted by fungal spores as they preferentially dug the infected substrate. Such an unexpected behaviour was also reported for *Mo. pharaonis* ant colonies that display a clear preference for infected sites when they migrate to a new nest [20]. Similarly, young queens of the ant *F. selysi* are attracted to nest sites contaminated with *Beauveria* and *Metarhizium* pathogens [19], although the latter are known to be responsible for a considerable rate of failures during colony foundation by soil-nesting species [19].

From a functional perspective, the seemingly suboptimal preference shown by some colonies for a substrate containing live entomopathogenic fungus may be explained in several ways. First, the fungal pathogen may have manipulated the ants by luring them with odour cues in order to increase its probability to contaminate the whole ant colony*.* However, host manipulation often results from a process of coevolution between the host and highly specialized parasites. This is not the case with *Metarhizium* fungus, which targets a broad spectrum of insect hosts [21]. Together with the strong selection pressure usually exerted on hosts to resist manipulation [37], a fungus-driven attraction of ants to infected substrate appears an unlikely phenomenon in our case*.* Second, while being potentially a sanitary challenge for the ants, the presence of fungi may also be a cue associated with suitable nesting sites, indicating favourable ecological conditions, such as humid and humus-rich soil. Finally, regardless of substrate contamination, the similar death rates observed in all colonies after 40 h of digging indicate that the amount of conidia present in the soil was not a lethal threat for the ants. Previous studies found that contacts with a pathogen at non-lethal doses reduce the susceptibility of individuals to later exposure to the same pathogen [42–44] or others [45]. Although still controversial in invertebrates [46,47], this process of ‘immune priming’ could trigger the upregulation of specific immune genes involved in antifungal responses [48,49]. By enhancing the survival of group members to a later pathogen challenge, immune priming may be an important physiological component of social immunity that increases fitness gain at the colony level. The observed preference of some ant colonies for infected sites could therefore be an adaptive strategy for the host that leads to a colony-wide ‘vaccination’ if all nest-mates come into contact with a low level of contaminated soil. In order to be beneficial, such a vaccination effect requires the probability of re-encountering the same pathogen to be high, which is the case here because *M. rubra* ants and *Metarhizium brunneum* fungus naturally occur in the same habitats [22,23].

Overall, we showed that *M. rubra* colonies are able to discriminate between substrates on the basis of their pathogenicity, displaying either avoidance or attraction to the contaminated substrate. Mechanisms that underlie such discrimination remain unclear but lead to anisotropic nest patterns, thereby demonstrating the key role of soil biotic factors in nest morphogenesis. The preference for fungal-infected soils seems to be a colonial trait and may be associated with factors that are beneficial to the colony. Further investigations are still needed to understand whether these two distinct nesting strategies are based on genetic factors, are due to different features of ants' nesting biotopes or the outcome from differences in life-history traits of ant colonies such as their previous exposure to entomopathogenic fungi.

To conclude, we found that the pathogen load of a digging substrate is a key factor of nest morphogenesis in ant societies. The presence of entomopathogenic spores in the soil does not alter the growth dynamics of excavated nests but makes their shape less isotropic with a few long galleries extending in the substrate. Quite unexpectedly, pathogen avoidance was not systematic as some colonies even showed the opposite preference of fungus-contaminated substrate. The relevance of this seemingly suboptimal preference remains to be investigated. The present study is a first report of pathogen-induced changes in collectively built nests, and more work is needed to understand this relatively unexplored area of disease defence in social insects.

## Data accessibility

Data used in this article are available in the Zenodo Digital Repository: https://zenodo.org/record/1095404#.Wij_1kriaM8.
